# Role of antibody engineering in generation of derivatives starting from MOv19 MAb: 40 years of biological/therapeutic tools against folate receptor alfa

**DOI:** 10.1093/abt/tbac026

**Published:** 2022-10-27

**Authors:** Barbara Frigerio, Matilde Montermini, Silvana Canevari, Mariangela Figini

**Affiliations:** Biomarkers Unit, Department of Applied Research and Technical Development, Fondazione IRCCS Istituto Nazionale dei Tumori, Milan 20133, Italy; Biomarkers Unit, Department of Applied Research and Technical Development, Fondazione IRCCS Istituto Nazionale dei Tumori, Milan 20133, Italy; Fondazione IRCCS Istituto Nazionale dei Tumori, Milan 20133, Italy; Biomarkers Unit, Department of Applied Research and Technical Development, Fondazione IRCCS Istituto Nazionale dei Tumori, Milan 20133, Italy

**Keywords:** antibody engineering, monoclonal antibodies, antibody-based therapeutics, CAR-T, folate receptor alpha

## Abstract

In the 1980s, we developed and characterized numerous murine monoclonal antibodies (MAbs) directed against human tumor-associated antigens. This mini review is focused on the generation of derivatives of an anti-folate receptor α (FRα) MAbs, named MOv19, exploiting the antibody-engineering progresses in the last 40 years. The FRα location on the luminal surface of proliferating epithelial cells, inaccessible to circulation, versus its over-expression in the entire surface of numerous carcinomas suggested a role for anti-FRα MAbs in the diagnosis and/or treatment of solid tumors. Presently, two MOv19 derivatives are in clinical trials: a chimeric resurfaced version in an antibody-drug conjugate format (SORAYA trial, 2022) and the murine scFv in a second generation chimeric antigen receptor, CAR-T (Phase Ia, 2021). MOv19 and its derivatives could be considered a relevant example that well-characterized anti-tumor murine Mabs and antibody engineering could be combined to generate useful therapeutic tools.

## Teaser

Statement of Significance: In the last 40 years, numerous derivatives of an anti-folate receptor α (FRα) monoclonal antibody (MAb), named MOv19, were generated exploiting the antibody-engineering progresses. The reported example highlights that combination of well-characterized anti-tumor murine MAbs and antibody/cell engineering enables the generation of useful therapeutic tools.

## INTRODUCTION

This mini review is focused on the role played by the antibody engineering progresses in the generation of anti-cancer therapeutic tools exploiting a well-characterized anti-tumor murine monoclonal antibody (MAb), named MOv19.

During the second half of the 1980s, two independent research groups developed hybridomas using the crude membrane of an ovarian cancer (OC) cell line [1] or total protein extracts from a choriocarcinoma cell line [2]. In the first case, two MAbs were selected (MOv18 and MOv19) and one MAb was identified using the other method of immunization (LK26). Binding assays showed that: (i) MOv18 and MOv19 [1] and LK26 [3] bound with high affinity to ovarian tumor cells; (ii) the three MAbs were all directed to the same 38–40 kDa glycoprotein, subsequently identified as folate receptor α (FRα) [4]; and (iii) MOv19 and LK26 recognized an overlapping epitope (unpublished data) which was different from that recognized by MOv18 [5].

The approaches applied for generation and use of MOv19 murine MAb, and the main study outcomes are summarized in Table 1.

**Table 1:** Generation and characterization of MOv19

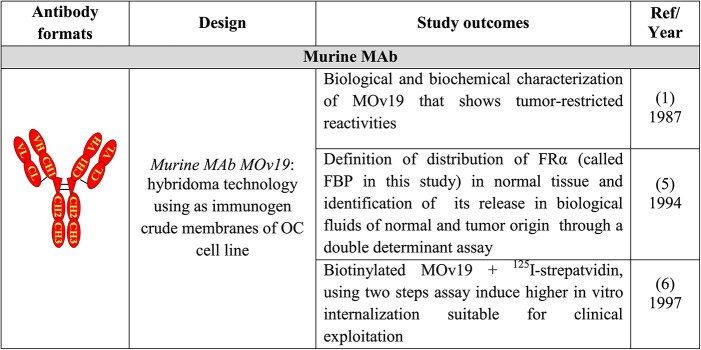

FRα is a membrane-bound glycosylphosphatidylinositol-anchored protein which is responsible for the folate transport in a variety of epithelial tissues. FRα is encoded by the *FOLR1* gene belonging to a family which include FRα and FRβ, membrane proteins, and FRγ, a soluble protein [6,7]. FRα is capable of transporting into the cell with high-affinity folic acid (KD < 10^−9^ M) and the physiologic circulating form of the vitamin, N5-methyltetrahydrofolate [8], but other folate uptake pathways, like the reduced folate carrier and the proton coupled folate transporter, are generally used by adult tissues [9,10]. FRα is over-expressed in multiple cancers, including ovary, breast, brain, lung, and colorectal cancer [11].

There are a number of unique advantages to exploiting FRα as a diagnostic/therapeutic target. In fact, FRα is located on the luminal surface of epithelial cells in most proliferating non-tumor tissues and is inaccessible to circulation; by contrast, FRα is expressed all over the cell in malignant tissue and is accessible via circulation. The role of the FRα in cancer development and progression and FR-targeted diagnostic/therapeutic tools have been reviewed [12,13].

## MAIN STEPS IN ANTIBODY ENGINEERING DEVELOPMENT

Antibody therapy was first proposed by Paul Ehrlich in 1900 as a magic bullet, but it takes until 1986 before the first antibody (Muronomab-CD3) was approved for the treatment of kidney transplant rejection. The first MAb for cancer therapy (Rituximab) was approved in 1997 by the Food and Drug Administration (FDA) for human use. This result became possible mainly thanks to the following biotechnological discoveries: hybridoma technology and polymerase chain reaction (PCR) along with sequencing and phage display antibody library.

In 1975, Köhler and Milstein invented the hybridoma technology (Nobel Prize in 1985), which allows for the production of large amounts of MAbs with a predetermined specificity [14,15].

In 1983, the second biotechnological advance was obtained by Kary Mullis who discovered PCR [16], a technique which would to revolutionize molecular biology (Nobel Prize in 1993). The modular arrangement of immunoglobulin domains associated with PCR and sequencing technique facilitated antibody engineering [17]. The gene segments encoding the domains of interest are isolated from the mRNA of a culture of hybridoma cells and are amplified by using PCR and cloned into expression vectors that contain genes encoding the human constant domains. In this way, by transplanting the domains of interest of a mouse antibody into a human antibody’s backbone, it was possible to build chimeric antibodies. The development of genetic engineering has been central to the clinical use of antibodies.

Humanized antibodies are created by grafting the complementarity-determining regions (CDRs) from a mouse’s MAb into a human’s IgG framework; unfortunately, since the frameworks of an antibody are fundamental for the correct tridimensional exposure of the CDRs, the generation of high-affinity humanized antibodies generally also requires the modification of additional residues in the frameworks. Several variants of the humanization technology have been developed [18].

Thanks to the modular organization of the antibody structures using PCR, it was also possible to amplify the variable-heavy (VH) and variable-light (VL) domains to develop functional antibody fragments. The most commonly used fragment is the single-chain Fv (scFv) composed of the VH and VL domains of the antibody and utilizes a flexible peptide linker to covalently join them in a single polypeptide [19]. ScFv, at present, is the most frequently used building block to create novel antibody-based reagents.

The ideal reagent for human therapy could be a completely human antibody. In 1990, John McCafferty, at the MRC in Cambridge, demonstrated that the expression on the surface of filamentous bacteriophages of an antibody fragment (FAb or scFv) was possible [20], thus opening the way to the construction of human libraries expressed on phage, leading to the selection and production of human antibodies without the need of employing animals.

A crucial advantage of the phage display technology is the linkage of the displayed antibody’s phenotype with its encapsulated genotype, which permits the rapid determination of the amino acid sequence of the specific binding antibody. There is a chance that the binders may result in a moderate affinity to the targets when panning naive libraries, and to overcome this limitation, a very large naive natural libraries should be used and this may be very laborious. Moreover, the lack of the natural pairings of heavy and light chains present in B cells could potentially lead to the generation of antibody molecules that have suboptimal biophysical characteristics that may cause problem such as aggregation, immunogenicity, and solubility.

Over the last four decades, different murine MAbs have been selected and well characterized; applying phage display and epitope imprinting selection, also defined as “guided selection,” [21–23] it is possible to obtain a human MAb with the same or overlapping epitope specificity using one of the mouse antibody chain (either VH or VL) as a template to drive selection. In the case of MOv19, the murine VL was paired with a human VH repertoire expressed on phage, and upon selection of the human VH, the latter was used to drive the selection of the human VL, obtaining a completely human antibody fragments able to compete with the parental antibody MOv19 [24]. The human derivative fragment has then be employed to develop other immunoreagents.

The parallel development of antibody engineering and of derivatives of MOv19 is summarized below in the next three sections.

## DERIVATIVES OF MURINE MAB MOV19

PCR amplification of the variable domains linked together in an scFv format generated the building block for the construction of: a fusion protein between the MOv19 scFv and interleukin-2 (IL-2) to improve tissue penetration for IL-2, reducing the toxicity related to the systemic administration of IL-2; a MOv19 intracellular scFv built to contain a KDEL retention signal to block the scFv in the endoplasmic reticulum to block the expression of the FR on cell surface to study its role in OC progression. The main outcomes of the relevant studies are summarized in Table 2.

**Table 2:** Generation and biological/clinical applications of the scFv MOv19

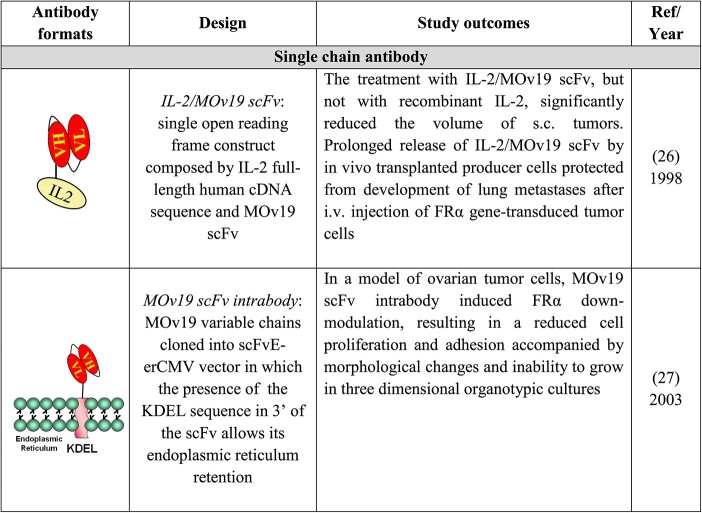

## CHIMERIC, HUMANIZED, AND HUMAN DERIVATIVES OF MOV19

With the purpose of reducing the immunogenicity of MOv19, its murine CL and CH γ2a genes were substituted with the genes encoding the human CL and CH γ1 constant regions [25]. The main study outcomes are summarized in Table 3 (“Chimeric antibody”).

**Table 3:** Preclinical and clinical development of the anti- FRα chimeric/resurfaced/human MOv19 derivatives

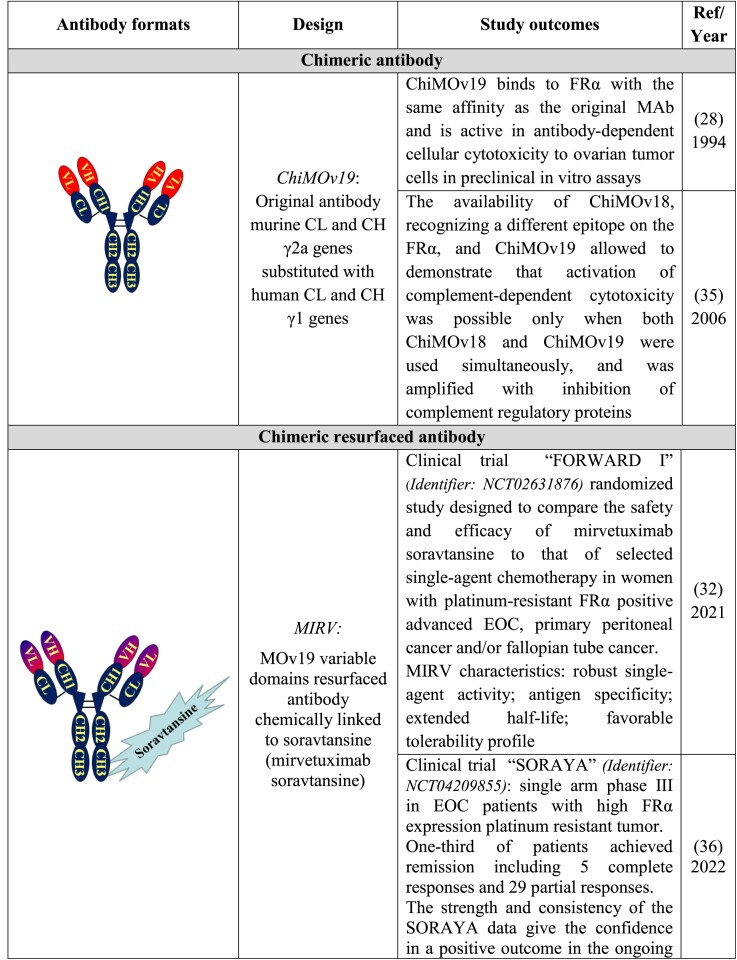
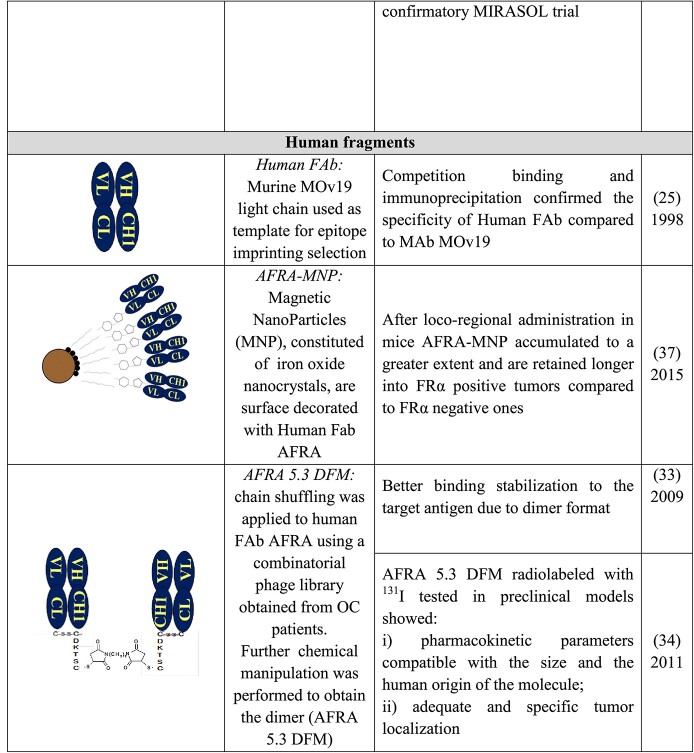

Subsequently, a chimeric resurfaced MOv19 [26,27] named M9346A, was used for linking soravtansine to generate IMGN853, an antibody drug conjugate (ADC) [28]. IMGN853, now named MIRV, was evaluated in the FORWARD I Phase III clinical trial; the conclusion [29] reported is: “In patients with platinum-resistant EOC, MIRV did not result in a significant improvement in progression free survival compared with chemotherapy. Secondary endpoints consistently favored MIRV, particularly in patients with high FRα expression. MIRV showed a differentiated and more manageable safety profile than chemotherapy.” The following clinical trial SORAYA supports the clinical impact of MIRV. On the basis of SORAYA results, ImmunoGen, on March 2022, announced the submission of a Biologics License Application under the accelerated approval of FDA for MIRV.

The first complete human FAb fragments against FRα [24] were produced using phage display and epitope imprinting selection [21], as described above. The binding affinity of the human FAb fragment (2 × 10^7^ M^−1^) was approximately fivefold weaker than that of the murine MOv19. Unfortunately, due also to its poor production yield, its development for in vivo clinical use was abandoned. To improve the FAb characteristics, a light chain library derived from OC patients was built and new selections using the chain shuffling approach were performed [24]. The obtained human FAb AFRA5 recognized FRα epitope overlapping that of the guiding reagent (VL of MOv19). AFRA5, after further optimization as chemical dimer, named AFRA-DFM5.3, was considered to be suitable for in vivo preclinical evaluation [30,31]. The main outcomes of the relevant studies are summarized in Table 3 (“Chimeric resurfaced antibody” and “Human fragments”).

## PRECLINICAL AND CLINICAL DEVELOPMENT OF MOV19 DERIVATIVES FOR LYMPHOCYTE ACTIVATION

In the last decades, scientists have studied methods to take advantage of T cell potency in cancer therapy by redirecting them against tumors independently from the T cell receptor-defined specificity. The promising initial approaches were based on two approaches. Bispecific antibodies (BsAbs), reagents that combine the specificities of two antibodies in a single molecule; and chimeric antigen receptor (CAR), synthetic immune receptors consisting of antigen-binding domains derived from an antibody and intracellular signaling domains derived from the T cell receptor. In this way, T cells expressing CARs redirect T cell to the tumor in a non-MHC-restricted manner. The highly modular nature of CAR-T therapeutics allows for an even greater product diversification, as was evident in the development of several different generations of CAR-T designs [32].

In the early 1990s, MOv19 was the first anti-cancer antibody utilized for generating—with an anti-CD16 MAb—a BsAb for solid tumors [33] to activate the NK cells. Subsequently, MOv19 was used to generate a BsAbs with specificity for TCR gamma/delta. Despite very encouraging results obtained in preclinical models, this approach was stopped due to its murine origin and due to the fact that the technology to purify the reagent was very complicated, thus very costly.

The main outcomes of the relevant studies are summarized in Table 4.

**Table 4:** Preclinical and clinical development of MOv19 and murine and human scFvs for lymphocyte activation

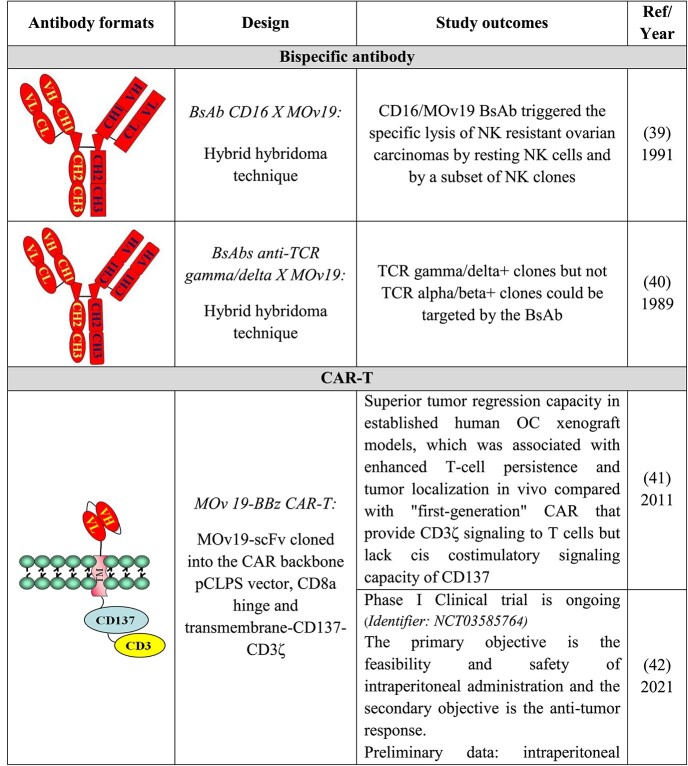
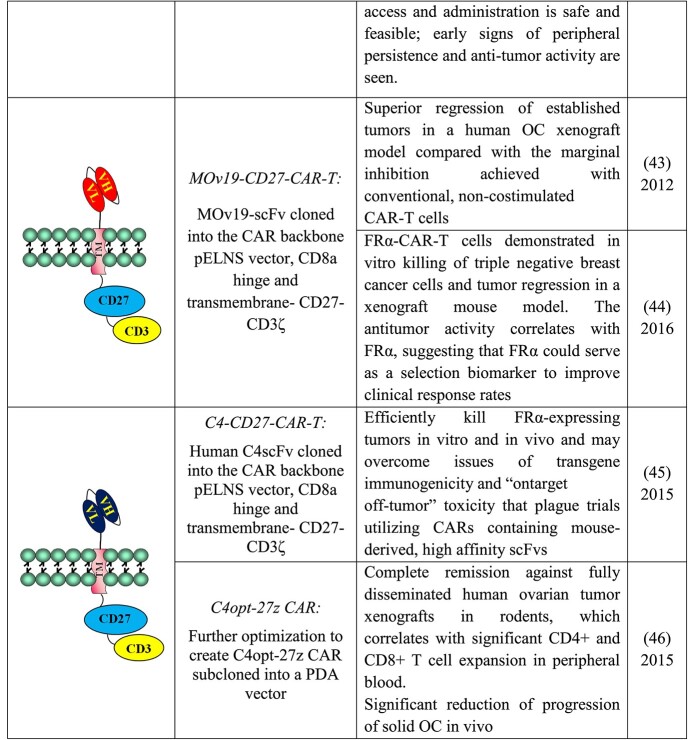

## DISCUSSION AND CONCLUSIONS

In 1986, OKT3, a murine anti-CD3 MAb, was the first to receive an approval from the FDA; and in 2021, the 100th MAb was approved [34]. At present, biologics account for approximately 25% of all drugs approved by the FDA, and in the last 5 years, MAbs are the most prevalent drug over all types; in 2021, 10 MAbs, 4 of them for cancer therapy, were approved [35]. In 2008, in a book [36] dedicated to Kohler and Milstein for their seminal work on antibody, the editors clearly introduced the concept that “design of a therapeutic antibody involves target selection, antibody generation, and engineering for optimal efficacy” [37]. As a relevant example, highlighting the importance of both antigen and MAb selection, emergence of targeted therapies such as the anti-HER2 humanized MAb trastuzumab (FDA approval in 1998), has been one of the most relevant advancements in the management of metastatic breast cancer. However, despite the evidenced efficacy, concurrent chemotherapy was still needed to maximize response and further approaches, as the use of ADC, were applied. In 2013, a humanized MAb directed against HER2 linked to a selected drug (ado-trastuzumab emtansine) revolutionized the field of ADCs as the first ADC approved by the FDA for the treatment of solid tumors; and in 2020, a second anti-HER2 ADC (trastuzumab-deruxtecan) was approved [38]. In the same decades, the combination of antibody and cells engineering enabled the development of CAR T cell therapy, which generated substantial excitement among researchers and oncologists; and since 2017, six CAR T cell therapies able to eradicate advanced leukemias and lymphomas have been approved by FDA for the treatment of blood cancers [39].

Our data, when considered in the context of clinical applications of anti-solid tumors MAbs and CAR-T, suggest that the two engineered derivatives of MOv19, Mirvetuximab and MOv19-BBz CAR-T, could be considered as useful therapeutics. Antibody engineering approaches have been applied to the other two anti-FRα antibodies (MOv18 and LK26) generated in the 1980s, and their derivatives entered in preclinical and clinical trials with promising results [11,12,40]. However, a direct comparison of their efficacy is not possible due to different investigated approaches.

Overall, the successful generation and application of antibody engineering to MOv19 in the last 40 years could be considered as another demonstration that combination of a well-characterized anti-tumor murine MAb with up-to-date antibody/cell engineering approaches could result in the generation of useful therapeutic tools.

## Data Availability

All data in this minireview are available and described in the references.
