# Correction to: Role of antibody engineering in generation of derivatives starting from MOv19 MAb: 40 years of biological/therapeutic tools against folate receptor alfa

**DOI:** 10.1093/abt/tbae010

**Published:** 2024-05-15

This is a correction to: Barbara Frigerio, Matilde Montermini, Canevari Silvana, Mariangela Figini, Role of antibody engineering in generation of derivatives starting from MOv19 MAb: 40 years of biological/therapeutic tools against folate receptor alfa, *Antibody Therapeutics*, Volume 5, Issue 4, October 2022, Pages 301–310, https://doi.org/10.1093/abt/tbac026

In the original publication there was an error in the third author's names which were transposed. The name should read in order: “Silvana Canevari”.

This error has been outlined only in this correction notice to preserve the version of record.

